# Retrospective Evaluation of Anesthetic Management in Cesarean Sections of Pregnant Women with Placental Anomaly

**DOI:** 10.1155/2020/1358258

**Published:** 2020-04-30

**Authors:** Aykut Urfalıoglu, Gözen Öksüz, Bora Bilal, Seyma Teksen, Feyza Calışır, Ömer Faruk Boran, Hafize Öksüz

**Affiliations:** Department of Anesthesiology and Reanimation, Kahramanmaras Sütcü İmam University Faculty of Medicine, Kahramanmaras, Turkey

## Abstract

**Background:**

In this study, patients who underwent cesarean section and had placenta previa and placenta accreta were examined and compared in terms of haemorrhagic indicators and perioperative anesthetic management.

**Methods:**

A retrospective study was conducted in a university hospital in Kahramanmaras, Turkey. It included 95 pregnant women who had placental anomaly and underwent cesarean section between December 15, 2014, and February 15, 2016.

**Results:**

The pregnant women were divided into two groups: Group P (previa) (*n* = 67) and Group A (accreta) (*n* = 28). The types of anesthesia administered were general anesthesia (GA), which was administered to 50 patients (74.6%) in Group P and 27 patients (96.4%) in Group A, and spinal anesthesia (SA), which was administered to 17 patients (25.4%) in Group P and one patient (3.6%) in Group A.. The mean blood loss was 685.82 ± 262.82 in Group P and 1582.14 ± 790.71 in Group A, and the given amount of crystalloid was higher in Group A with an average of 1628.57 ± 728.19 ml. The use of erythrocyte and fresh frozen plasma solution was higher in Group A than Group P. Eleven patients were intubated and taken to the Intensive Care Unit (ICU) in Group A. Postoperative mechanical ventilation duration was significantly higher in Group A (75.14 ± 43.84 h) (*p* < 0.001). ICU stay was longer in Group A with 2.80 ± 1.13 days. (*p* < 0.001).

**Conclusion:**

The intraoperative management and the availability of postoperative ICU conditions are important in placental anomalies cases. The communication between operation team with regard to the development of a standard protocol for these cases will be of great benefit in reducing morbidity and mortality.

## 1. Introduction

Peripartum hemorrhage (PPH) is the third most common reason for maternal deaths in the United Kingdom at a rate of 10%; in the United States, this rate is 11.4%, and in Asia and Africa, it is 30% [1–3]. Despite the low incidence of placenta previa (PP) and placenta accreta (PA) (0.5% and 0.3%, respectively), peripartum hemorrhage may occur [4, 5]. The definition of previa states that, in addition to normal localization of the placenta, there is complete or partial coverage of the uterus internal cervical os, and it has been reported as common in cases of multiparity, maternal age of over 35 years, cigarette smoking, and infertility treatment [6, 7]. Placenta accreta is defined as the invasion of the placenta decidua within the myometrium and is named placenta increta in cases of deepening invasion and as placenta percreta when it goes beyond the uterus. Thus, the term PA is usually used to explain these three conditions [8]. Although the standard definition of major obstetric hemorrhage (MOH) is hypovolemia obscured both with physiological signs and with hemodilution formed with amniotic fluid, it may also be defined as >1500 ml to >3000 ml in less than three hours or 150 ml/min in 20 min blood loss and the need for >4 units of acute blood transfusion [9, 10]. The most recent definition is erythrocyte suspension (ES) of >10 units in 24 hours or four units in one hour [11]. Intraoperative bleeding can also be included in PPH, which is defined as hemorrhage seen within 24 hours after birth [12]. Because acute massive hemorrhage (which can occur due to placental anomalies) may increase maternofetal mortality, intraoperative and surgical management of these pregnant women should be performed appropriately.

In this study, a retrospective analysis of perioperative anesthetic management was performed in cesarean sections of pregnant women diagnosed with placental anomalies in our hospital. Thus, an internal audit was conducted in our clinic to determine the deficiencies in the practices related to these cases and to take the necessary preventive precautions.

## 2. Materials and Methods

### 2.1. Study Design and Setting

All procedures performed in studies involving human participants were in accordance with the ethical standards of the institutional and/or national research committee and with the 1964 Helsinki declaration and its later amendments or comparable ethical standards. From the archived records and files, a cross-sectional retrospective study was made of cases who underwent cesarean section because of a diagnosis of placental location and invasion anomaly between December 15, 2014, and February 15, 2016.

Patients diagnosed with PP were designated as Group P and those with PA as Group A. An examination was made of the demographic data of the patients, the level of emergency of the operation, anesthetic method and interventional procedures, estimated amount of blood loss, maternal preoperative and postoperative Hb values, arterial blood gas pH of patients, given blood products, lactate values, outcome of operation, admittance to intensive care unit (ICU), and duration of mechanical ventilation (MV). In addition, estimated intraoperative blood loss and the use of crystal, colloid, erythrocyte suspension (ES), and fresh frozen plasma solutions (FFP) were investigated.

### 2.2. Statistical Analysis

Taking the study of Seyhan Özkan et al. [13] as a standard, an *α* value of 0.05 and power of 0.95 was used. Analyses of the data obtained in the study were made using Statistical Package for the Social Sciences (SPSS 22.0) software. Conformity of the data to normal distribution was assessed using the Shapiro–Wilk test and variance homogeneity, with Leneve's test. In the comparison of the two independent groups, the independent samples *t*-test was used together with bootstrap results. Quantitative data were presented in the tables as mean ± standard deviation (SD) and median (minimum-maximum) values, and categorical data were stated as number (*n*) and percentage (%). A value of *p* < 0.05 was accepted as statistically significant.

## 3. Results

The ninety-five patients were determined to have undergone surgery with a diagnosis of placental location and invasion anomalies. The PP group (Group P) had a mean age of 30.26 ± 5.37 years, and the PA group (Group A) had a mean age of 32.35 ± 4.85 years. In Group P, 14 (20.9%) cases were primiparity, and, in Group A, all the cases (100%) were multiparity. The operating time for Group A patients was determined to be significantly longer (*p* < 0.001) (Table 1). A total of 20 (29.9%) of the 67 patients in Group P and 15 (53.6%) of the 28 patients in Group A were admitted for an emergency operation. The types of anesthesia administered were general anesthesia (GA), which was administered to 50 patients (74.6%) in Group P and 27 patients (96.4%) in Group A, and spinal anesthesia (SA), which was administered to 17 patients (25.4%) in Group P and one patient (3.6%) in Group A. The anesthetic interventions are depicted in Table 2.

**Figure 1:**
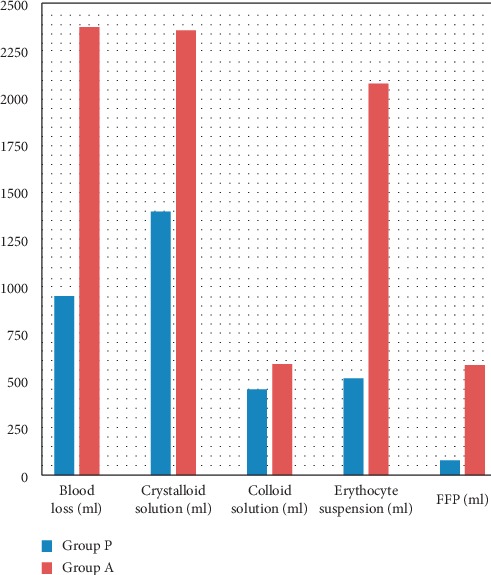
Data related to the intraoperative estimated blood loss and bleeding management of patients.

The estimated intraoperative blood loss and data related to the management of bleeding are displayed in Figure 1. The mean estimated blood loss was statistically significantly higher in Group A (1582.14 ± 790.71 ml) than in Group P (685.82 ± 262.82 ml), with *p* < 0.001. The crystalloid and colloid solutions administered to patients were 1098.50 ± 297.20 and 188.85 ± 262.07 in Group P and 1628.57 ± 728.19 and 267.85 ± 318.62 in Group A, respectively. The difference between the two groups was significant only in respect to the use of crystalloid (*p* < 0.001). Similarly, the use of ES and FFP was significantly higher in Group A (1189.35 ± 884.7 and 300 ± 280, respectively; *p* < 0.001) than in Group P (154.35 ± 355.05 and 14.8 ± 63.2, respectively; *p* < 0.001).

The laboratory and clinical data of the patients at different stages of the operations were investigated. At the end of the operation, 65 (97%) patients in Group P were extubated, and, in Group A, 17 (60.7%) patients were extubated, and 11 (39.3%) intubated patients were transferred directly to ICU. In patients with a requirement for postoperative MV, the duration of MV in Group A (75.14 ± 43.84 h) was significantly longer than in Group P (4.74 ± 20.78 h; *p* < 0.001). The mean length of stay in ICU was longer in Group A at 2.80 ± 1.13 days (*p* < 0.001) (Table 3).

## 4. Discussion

In this retrospective examination, the amount of bleeding was found to be greater in PA patients compared to PP patients, and in association with this finding, there was a higher rate of both fluid and blood product usage. It was also observed that a higher number of patients with PA had postoperative requirement for ICU, and their length of stay in ICU was longer.

In guidelines prepared for PPH, the subjects of the anesthesia method to be selected, fluid management-hemodynamic monitorization, blood product transfusion, laboratory evaluation, surgical methods, and postoperative follow-up have all been considered in obstetric hemorrhage [9, 12, 14, 15]. Through correlation with the guidelines, these procedures are applied to patients with placental anomalies. The increased bleeding which forms rapidly during the operation because of the anatomic and physiological characteristics of the uterus and placenta in these cases may lead to death with shock and organ failure [16].

Even though it is known that some patient-related demographic factors increase pregnancy-associated placental anomalies, it has been reported that previous uterine surgery and multiple cesarean deliveries lead to decidua defects, in particular, increasing the frequency of these cases [8]. In some cases undergoing cesarean because of a preoperative PP diagnosis, PA may be seen in postoperative tissue examination [7]. Therefore, as recommended by the Royal College of Obstetricians and Gynecologists (RCOG), ultrasound examination should be made in the twentieth antenatal week and later in suspicious cases, and when necessary, follow-up with magnetic resonance imaging (MRI) should be applied every three to four weeks, especially in the presence of PA [14, 17].

In pregnancies with placental anomalies, although the standard delivery date is generally the thirty-fourth gestational week, Silver and Barbour [8] recommend 34 to 36 weeks in stable cases and 32 to 34 weeks as appropriate in cases with a history of bleeding and delivery before 32 weeks only in emergency cases. In the obstetrics clinic of our hospital, cases without problems are generally kept until the thirty-sixth week. In the cases examined in this study, the mean gestational week of the PP cases was higher than that of the PA cases, and when cases which delivered before the thirty-second week were examined, more PA cases were observed. This high rate could be associated with more complications seen in PP than PA, admittance under emergency conditions at a higher rate, or the presence of either patient- or physician-related problems in the antenatal diagnosis.

General anesthesia is preferred in cesarean operations in most cases with placental anomalies. This preference can be explained by the increased tendency for bleeding with the loss of clotting factors and rapidly deteriorating hemodynamics in a short time; furthermore, hemodynamics can be worsened with sympathetic blockage in regional anesthesia, with an increased risk of hematoma. In addition, bleeding and increased surgical manipulation prolong the operating time, and these are other factors causing discomfort in an awake patient [13, 18, 19]. Nevertheless, in a questionnaire applied to obstetric anesthetists, it was reported that 65% applied SA to PP cases where complications are not expected, and GA was preferred by 69.2% in cases with suspected PA and by 96.2% in cases diagnosed with PA. Gunaydın et al. have reported successful management with SA in patients with PP and PA [20]. In the current study, SA was applied to PP cases where fewer complications were expected and GA was preferred for the other PP cases and all but one of the PA cases. Although GA was more preferred, unlike previous reports in literature, the lower preference for SA in the current study can be explained by antenatal monitoring and diagnosis using ultrasound being not sufficiently reliable and the admission of more PA cases under emergency conditions.

Coagulopathy that forms with the destruction of clotting factors and unstable hemodynamics makes patient management more difficult [21]. The RCOG recommends that, in cases where major hemorrhage is expected, standard monitorization should be started with two 14-gauge peripheral cannulas, and then intra-arterial pressure (IAP) monitorization should be added, and, if necessary, central venous cannulation intervention [12]. The records revealed that the vast majority of the current study cases diagnosed with PP were not applied with catheter intervention, and in 75% of the PA cases, arterial and central venous interventions were made together.

In cases of MOH, a rapid infusion of 3.5 L of warmed fluid, primarily 2 L of crystalloid until blood is protected, is recommended [12]. Despite the higher rates of application of crystalloid infusion to PA cases, the amount was not as high as in the guidelines. This was due to the use of crystalloid preventing dilutional coagulopathy adding to the loss of factors occurring in bleeding [22]. Although there are no specific criteria related to the starting time of ES infusion, it is recommended that it is applied according to the clinical and laboratory data of the patient [15]. Similarly, the subject of when and at what rates blood products such as FFP and thrombocytes should be applied remains a matter of debate. Although different rates have been mentioned, the most recent recommendations suggested that following four units of ES, four units of FFP (12–15 mg/kg) should be given at the ratio of 1 : 1 and that if platelet count <75 × 10^9^/L, one unit of thrombocyte infusion should be given [12]. As expected, in the current study, ES and FFP were utilized at higher rates in PA cases where there were more surgical procedures and longer operating times. However, rather than transfusion being made at a fixed rate, ES and FFP were administered according to the intraoperative hemodynamic characteristics of the patients in both PP and PA cases. In cases of severe bleeding, when the hemodynamic status was rapidly deteriorating, ES and FFP were administered at varying times and rates, but generally early. Thrombocyte solution was not applied routinely intraoperatively but only as necessary according to the thrombocyte results in the postoperative period. It has been reported that the aim of transfusion in obstetric hemorrhages is to provide sufficient organ perfusion and oxygenation with fluid and blood transfusion and to treat or prevent the formation of coagulopathy associated with massive blood loss developing in these patients [23].

Laboratory data are of great importance in respect to the timing and maintenance of intraoperative transfusion, the need for supportive treatment, and follow-up of the clinical status. Examination of coagulation parameters, such as hemogram, PT, a-PTT, INR, and fibrinogen, is recommended [15, 24]. However, only blood gas analysis can be preferred instead of these classical parameters.

When the pre- and postoperative arterial blood gas values were examined, no difference between the groups in respect of Hb, pH, and lactate values was observed. This result may suggest that the cases were well managed intraoperatively. Cross-matching of blood types must be applied in elective cases, and in emergency cases, O Rh (-) ES can be applied without waiting for the 60 min required for cross-matching [15, 25, 26]. In our clinic, four cross-matched units, each of ES and FFP, are prepared routinely in elective cases, and in severe cases, these products at a greater rate together with fibrinogen suspension are prepared and made available in the operating room. In emergency cases, blood preparation is made rapidly with a sample for testing, and until it is provided, O Rh (-) ES is applied if necessary.

At the end of the operation, patients with no severe bleeding or impaired hemodynamics were extubated, whereas those with unstable hemodynamics and poor arterial blood gas values were transferred to ICU while being intubated. In the current study, rates of intubation were higher and length of stay in ICU was longer in the PP patients in Group A. This could be attributed to more bleeding in Group A patients (as anticipated), higher transfusion rates, lower body temperature, and greater surgical manipulation.

Some limitations need to be considered in explaining our findings. This study represented a proper investigation in the field of obstetric anesthesia; however, since coagulation problems were often observed in these cases, the absence of fibrinogen examination can be considered a serious limitation. In MOH, fibrinogen has been shown to be the best marker, and the early application of fibrinogen and the maintenance of fibrinogen levels at >2 g/L is considered to be of extreme importance [12, 24, 25].

## 5. Conclusion

The results of this study demonstrated that there could be a need for several anesthetic and surgical interventions in cases with placental anomalies, primarily PA, which can cause severe obstetric hemorrhage in cesarean operations. The elimination of deficiencies in this subject will provide significant benefit and can be achieved by developing an appropriate protocol, including intraoperative and postoperative management of the patient, especially intraoperative fluid management and communication with the blood bank, and ensuring the adequacy of postoperative ICUs.

## Figures and Tables

**Table 1:** Demographic data of patients.

	Group P (*n* = 67)	Group A (*n* = 28)	*pvalue*
Age (years)	30.26 ± 5.37	32.35 ± 4.85	0.079
BMI (kg/m^2^)	29.54 ± 3.92	28.95 ± 3.98	0.507
Gestational week (w)	35.82 ± 3.53	33.35 ± 5.43	**0.010** ^*∗*^
Operation time (minute)	52.43 ± 13.34	80.53 ± 26.02	**<0.001** ^*∗*^

^*∗*^ *p* < 0.05: significant statistical value. Quantitative data are presented as mean ± standard deviation and median value (min-max). Categorical data are presented as *n* (number) and percentage (%). BMI : body mass index.

**Table 2:** Anesthetic and surgical procedures applied to patients in operation.

		Group P (*n* = 67) (%)	Group A (*n* = 28) (%)	*pvalue*
Type of operation	Emergency	20 (29.9)	15 (53.6)	**0.026** ^*∗*^
	Electively	47 (70.1)	13 (46.4)	
Type of anesthesia	General	50 (74.6)	27 (96.4)	**0.009** ^*∗*^
	Spinal	17 (25.4)	1 (3.6)	
Catheter applications	Patients who need IAP monitoring	18 (26.9)	4 (14.3)	**0.001** ^*∗*^
	Patients who need CVP + IAP monitoring	3 (4.5)	21 (75)	

^*∗*^ *p* < 0.05: significant statistical value. Quantitative data are presented as median value (min-max). Categorical data are presented as *n* (number) and percentage (%). IAP : intra-arterial pressure; CVP : central venous pressure.

**Table 3:** Laboratory and clinical data on patients and newborns at different stages of operation.

	Group P (*n* = 67)	Group a (*n* = 28)	*pvalue*
Preoperative Hb (g/dl)	11.51 ± 1.18	11.56 ± 1.28	0.861
Postoperative Hb (g/dl)	10.40 ± 1.39	10.31 ± 1.32	0.844
Postoperative pH	7.48 ± 0.50	7.55 ± 1.12	0.835
Postoperative lactate (mmol/L)	2.00 ± 2.18	1.86 ± 1.72	0.828
MV time (h)	4.74 ± 20.78	75.14 ± 43.84	**<0.001** ^*∗*^
ICU duration of stay (day)	2.25 ± 0.50	2.80 ± 1.13	**<0.001** ^*∗*^

^*∗*^ *p* < 0.05: significant statistical value. Quantitative data are presented as mean ± SD (standard deviation) and median value (min-max). Categorical data are presented as *n* (number) and percentage (%). Hb : hemoglobin; MV : mechanic ventilation; ICU : intensive care unit.

## Data Availability

The datasets used and/or analyzed during the current study are available from the corresponding author on reasonable request.
